# Patterns and outcomes of real-world high-flow nasal cannula use: a multi-hospital retrospective cohort study

**DOI:** 10.62675/2965-2774.20260366

**Published:** 2026-02-20

**Authors:** Diana C. Bouhassira, Chad H. Hochberg, Sarina K. Sahetya, Ann Parker, Khyzer B. Aziz, Li Yan, Theodore John Iwashyna

**Affiliations:** 1 Johns Hopkins University - Baltimore School of Medicine Department of Medicine Maryland United States Division of Pulmonary and Critical Care, Department of Medicine, School of Medicine, Johns Hopkins University - Baltimore, Maryland, United States.; 2 Johns Hopkins University - Baltimore School of Medicine Department of Pediatrics Maryland United States Division of Neonatology, Department of Pediatrics, School of Medicine, Johns Hopkins University - Baltimore, Maryland, United States.

**Keywords:** High flow nasal cannula, Respiratory therapy, Airway extubation, Acute respiratory failure, Hypoxemia, Critical care, Intubation, Patient discharge, Intensive care units

## Abstract

**Objective:**

To characterize real-world high-flow nasal cannula use and outcomes.

**Methods:**

A retrospective observational study using an electronic health record registry of all adult patients in a five-hospital system between 2017 - 2025. We identified all high-flow nasal cannula episodes, defined as periods of high-flow nasal cannula use containing breaks no longer than 6 hours. We describe key measurements, including high-flow nasal cannula episode duration, intubation rates, and death or hospice discharge rates; secondary outcomes included intensive care unit admission, lengths of stay, and discharge location. We used adjusted hierarchical logistic regression to evaluate variation across hospitals.

**Results:**

28,269 high-flow nasal cannula episodes from 17,519 individual patients over 19,313 hospitalizations were identified. Average high-flow nasal cannula use increased from 176 episodes/month in 2017 to 269 episodes/month by 2024 (p < 0.001). Median episode duration was 13.9 [interquartile range 4.3 - 36] hours, 24.5% of episodes were followed by intubation, and 29.3% by death or hospice discharge; 83.6% of high-flow nasal cannula use was escalation respiratory therapy, with the remainder used within 24 hours of extubation. Illness severity, admitting service, hospital, and unit type were associated with the odds of intubation and the duration of high-flow nasal cannula episodes; 16% of the variation in mortality and 20% of the variation in intubation were attributable to hospital-level variation.

**Conclusion:**

High-flow nasal cannula is used in multiple hospital settings and contexts. The use of high-flow nasal cannula has increased between 2017 and 2025. Outcomes of high-flow nasal cannula use are influenced by both patient characteristics and the contexts in which it is used.

## INTRODUCTION

Over the last two decades, high-flow nasal cannula (HFNC) has emerged as an important treatment for respiratory failure.^(1-4)^ Physiologic studies show that HFNC provides a consistent, high fraction of inspired oxygen and some modest positive pressure, reduces ventilatory dead space, and favorably influences lung mechanics.^(5-9)^ Randomized clinical trials comparing HFNC to other modalities have shown mixed results, but have suggested improvement in clinical outcomes in patients with acute hypoxemic respiratory failure (AHRF) and after extubation.^(10-15)^ Practice guidelines now encourage the use of HFNC, and it has been incorporated in the updated global definition of acute respiratory distress syndrome.^(16-18)^ Compared to other advanced respiratory support devices, HFNC is perceived as easier to initiate and requires less sedation.^(19-21)^

Despite growing enthusiasm for HFNC, generalizable data on real-world use and outcomes in the last decade of clinical practice are limited.^(22-24)^ This is important because, while clinical trials provide a basis for understanding HFNC use in select patient populations, trial participants often differ meaningfully from non-participants in terms of demographics and illness severity.^(25)^ Hence, complementary strategies are needed to measure which patients are being treated with HFNC and their outcomes, among the general adult inpatient population.

In this study, we utilized an electronic health record (EHR) registry of 17,519 adult patients treated with HFNC in a multi-hospital system over the last 8 years to fill this knowledge gap. Our objective was to characterize real-world HFNC use and outcomes.^(26-28)^ These data can help place existing trials in context and support the development of future trials.

## METHODS

### Study design

The Johns Hopkins Institutional Review Board approved this protocol with an informed consent waiver (IRB00391128). We included all patients aged 18 years or older who started HFNC for any reason between July 1^st^, 2017, and March 1^st^, 2025, at two academic centers and three community hospitals comprising a hospital system in the United States.

An HFNC episode was any period during which a patient was documented as receiving HFNC. When a patient had an interruption in HFNC use lasting ≤6 hours, we considered the periods of HFNC use preceding and following the interruption as continuous, and treated them as a single HFNC episode; otherwise, we considered them as two distinct HFNC episodes. The 6-hour gap allowed for patients alternating between different forms of non-invasive respiratory support, and is concordant with recent trial work allowing 6 hours of non-invasive positive pressure ventilation (NIPPV) prior to randomization.^(11)^

We categorized HFNC episodes as "post-extubation" if HFNC was initiated within 24 hours after extubation, with the remainder characterized as "escalation" therapy.

For the primary clinical outcome, we assessed a composite of in-hospital mortality or hospice discharge. As covariates, we extracted relevant patient, illness, and hospitalization characteristics (details in Section 1S - Supplementary Material). ROX Index for Intubation, a risk index for progressing to intubation for patients on HFNC, was calculated based on the closest available respiratory rate and fraction of inspired oxygen (FiO_2_) after HFNC initiation, with ≥ 5 categorized as "low risk" and < 5 as "high risk".^(29,30)^ Respiratory failure was labeled as having a component of hypercapnia if associated with an arterial blood gas of pH of < 7.35 and a partial pressure of arterial carbon dioxide (PaCO_2_) > 45 drawn in the 24 hours prior to or 2 hours after HFNC initiation.^(11,14)^ Hospitalization characteristics included admitting hospital, unit type where HFNC was initiated, and clinical service. Hospital units with multiple bed types (i.e., intermediate care and ward beds) were categorized by the highest level of care provided.

**Figure 1:**
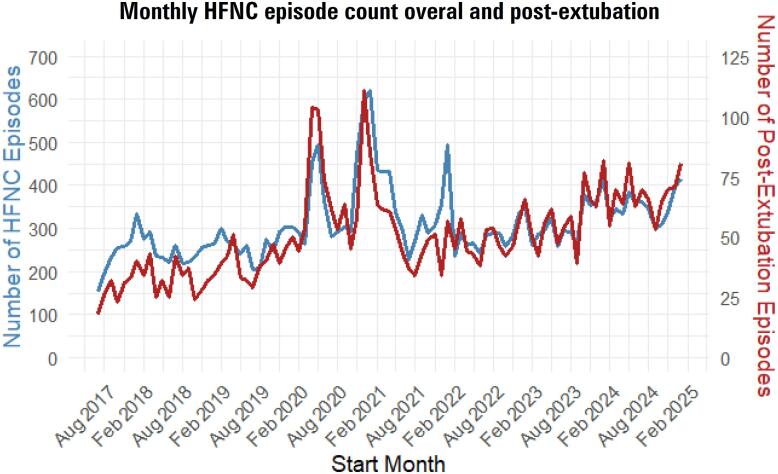
Trends in overall monthly high flow nasal cannula episode count and monthly number of post-extubation high flow nasal cannula episodes.

We removed HFNC episodes with implausible or missing values for key covariates and restricted our cohort to the first five HFNC episodes within a hospitalization (Figure 1S - Supplementary Material).

This report conforms to the Strengthening the Reporting of Observational Studies in Epidemiology (STROBE) guidelines.^(31)^ Analyses were performed with R version 4.4.2 (R Foundation for Statistical Computing, Vienna, Austria). Analytic code is available on GitHub (https://github.com/dbouhas1/CCS_HFNCManuscript).

### Statistical analysis

Our primary analyses focused on the whole cohort and a stratified analysis considered HFNC as escalation therapy *versus* post-extubation. For all exploratory models, standard errors were calculated accounting for clustering at the patient-level and we adjusted for pre-specified confounders: patient age, sex, race, ethnicity, Elixhauser comorbidity weighted mortality index, number of Elixhauser comorbidities, Sequential Organ Failure Assessment (SOFA) score the day of admission and day of HFNC initiation, ROX Index for Intubation (ROX) at HFNC initiation, coronavirus disease 2019 (COVID-19) status, admitting hospital type, clinical service, initiating unit type, and admission month.

Descriptive statistics of demographics, physiologic, and institutional variables are presented. To evaluate for changes in HFNC use before and after the COVID-19 pandemic, we compared the average monthly number of HFNC episodes, number of post-extubation HFNC episodes, proportion of HFNC episodes ending in death or intubation, and average HFNC episode duration in July - December 2017 *versus* July - December 2024, adjusting for monthly average patient age, Elixhauser comorbidity weighted mortality index, SOFA at HFNC initiation, and ROX at HFNC initiation.

A Sankey diagram was created to visualize the relative proportions of episodes across outcomes. We calculated and plotted the relative proportions of HFNC outcomes at 12-hour intervals over the first 96 hours after HFNC initiation. Using multivariable linear regression, we assessed factors associated with the duration of HFNC episodes in the overall cohort. Coefficients were interpreted as the average increase in duration in hours.

When analyzing intubation after HFNC, we considered episodes in which code status at HFNC initiation permitted intubation. We examined factors associated with the odds of intubation using multivariable logistic regression and those associated with the duration of HFNC episodes prior to intubation using multivariable linear regression. We further examined the *a priori* unknowable subset of episodes ending in intubation within 48 hours of HFNC episode end time; here, we excluded HFNC episodes initiated within 1 hour of mechanical ventilation start time and in which mechanical ventilation start time was followed by death within 1 hour.

To assess the impact of hospital effects relative to patient and illness characteristics, we fit multivariable mixed-effects logistic regression models for mortality and intubation, with a random-effects term for hospital. We calculated the predicted intubation and death rate across hospitals for a patient with average characteristics using parametric bootstrapping to obtain 95% confidence intervals (95%CIs).

## RESULTS

We identified 28,269 HFNC episodes from 17,519 individual patients over 19,313 hospitalizations. Median patient age was 67.5 [56.1 - 77.3] years (Table 1); 9,520 (54%) were male; 10,624 (61%) identified as white, 4,766 (27%) as black, and 768 (4%) as Hispanic. The patients had a median of 8 [5 - 11] Elixhauser comorbidities. Of 17,519 patients, n = 5,094 (29%) had more than one episode of HFNC.

**Table 1:** Characteristics of patients and high-flow nasal cannula episodes

Patient characteristics (n = 17,519)		
Age		67.5 [56.1 - 77.3]
Male		9,520 (54)
Race		
	Asian	817 (5)
	Black or African American	4,766 (27)
	White	10,624 (61)
	Other	1,206 (7)
	Unknown	106 (1)
Ethnicity		
	Hispanic	768 (4)
	Non-Hispanic	15,417 (88)
	Unknown	1,334 (8)
Preferred language		
	English	16,447 (94)
	Spanish	485 (3)
	Other < 100 speakers	581 (3)
ECI number of comorbidities		8 [5 - 11]
ECI (Weighted Mortality Index)		23 [6 - 41]
Comorbidities		
	Chronic lung disease	6,885 (39)
	Congestive heart failure	7,665 (44)
	Diabetes (complicated)	5,891 (34)
	Renal failure (moderate or greater)	7565 (44)
**HFNC episode characteristics (n = 28,269)**		
Hospital		
	A	13,035 (46)
	B	4,371 (15)
	C	4,685 (17)
	D	1,899 (7)
	E	4,279 (15)
Episode number		
	1	19,205 (68)
	2	5,313 (19)
	3	2,160 (8)
	4	1,040 (4)
	5	551 (2)
Day of SOFA		4 (3, 7)
ROX Index for Intubation at initiation		
	High risk	5,268 (25)
	Low risk	16,168 (75)
PaO_2_ at episode start		80 [64 - 108]
COVID positive		3,684 (13)
Preceding code status		
	CPR - full code	20,481 (72)
	No CPR - do NOT intubate	3,240 (1 1)
	No CPR - intubate	678 (2)
	No CPR - palliative and supportive care	351 (1)
	None listed	3,505 (12)
Most recent prior respiratory support		
	Face mask	6,536 (23)
	Mechanical ventilation	2,783 (10)
	Low-flow nasal cannula	11,598 (41)
	NIPPV	5,172 (18)
	No documented support	723 (3)
	Room air	1,454 (5)
Less than 24 hours after extubation		4,395 (20)
Location Initiated		
	Emergency Department	3,645 (13)
	Intensive care unit	19,277 (68)
	Intermediate care unit	641 (2)
	Procedural/labor & delivery suite	501 (2)
	Ward	4,205 (15)
Responsible service		
	Medical	19,435 (69)
	Surgical	5,076 (18)
	Other	3,758 (13)

ECI - Elixhauser Comorbidity Index; HFNC - high flow nasal cannula; SOFA - Sequential Organ Failure Assessment; PaO_2_ - arterial partial pressure of oxygen; CPR - cardiopulmonary resuscitation; NIPPV - non-invasive positive pressure ventilation. High-flow nasal cannula episodes are defined as continuous periods during which a patient is documented as receiving high-flow nasal cannula with no intervening alternate form of respiratory support lasting more than 2 hours. Results expressed as median (interquartile range), categorical variables as n (%).

### High-flow nasal cannula initiation

19,277 (68%) episodes were started on a unit with intensive care unit (ICU) capacity, 3,645 (13%) in an emergency department (ED), 641 (2%) in an intermediate care unit (IMC), and 501 (2%) in a procedural or labor & delivery (L&D) suite; 4,205 (15%) began outside of critical care spaces on the wards. Most HFNC episodes occurred in patients cared for by a medical service, with surgical services accounting for 5,076 (18%) episodes.

Most HFNC use was escalation therapy, with only 4,633 (16%) episodes occurring within 24 hours of extubation. Post-extubation HFNC use was less common in medical patients than in surgical patients (17% *versus* 27%; p < 0.001). Of escalation episodes, the prior mode of support was a low-flow nasal cannula (10,537; 45%), a face mask (6,025; 25%), or NIPPV (4,732; 20%).

Overall, median time from admission to first HFNC use was 3.34 [0.88 - 8.51] days. Patient code status documented prior to HFNC initiation explicitly precluded intubation in 3,591 (13%) episodes. ROX at HFNC initiation suggested a high risk of HFNC failure in 5,268 (25%) episodes and a low risk in 16,168 (75%) episodes. Of the 11,670 (41%) episodes associated with an arterial blood gas, the median partial pressure of arterial oxygen (PaO_2_) was 80 [64 - 108], median PaCO_2_ was 40 [34 - 46], and 1,345 (13%) suggested hypercapnic respiratory failure (nearly all with concomitant hypoxemia). Median PaO_2_/FiO_2_ (P/F) ratio was 148 [100 - 226] among those with a qualifying arterial blood gas; among the remainder, median peripheral oxygen saturation (SpO_2_)/FiO_2_ ratio was 157 [118 - 192].

Examining trends over time, monthly HFNC episodes increased from 175.7 episodes/month, including 25.5 episodes/month after extubation, in early 2017 to 268.7 episodes/month overall and 62.0 episodes/month after extubation by late 2024 (p < 0.001, respectively) (Figure 1).

HFNC - high flow nasal cannula.

### High-flow nasal cannula duration

Overall, HFNC was used for a median duration of 13.9 [4.33 - 36.0] hours. The distribution was right-skewed, with 75% of episodes lasting 36.0 hours or less, and the top 10% lasting 75.3 hours or more. Median duration of HFNC in escalation episodes was significantly shorter than in post-extubation episodes (13.0 [4.08 - 36.1] *versus* 17.5 [6.07 - 35.0] hours (p < 0.001)).

The duration of the HFNC episode was significantly associated with several patient, illness, and hospitalization characteristics. Factors which were associated with at least 6 additional hours of HFNC were: COVID-19 positive status (12.1 hours longer; 95%CI 10.0 - 14.1 hours), being located on the wards (9.6 hours; 95%CI 7.2 - 12.0 hours) or IMC (8.3 hours, 95%CI: 3.8 - 12.8 hours) at time of initiation. Full regression results are in table 1S (Supplementary Material). The mean duration of HFNC episodes did not change between 2017 and 2024.

### High-flow nasal cannula outcomes, including intubation

**Figure 2:**
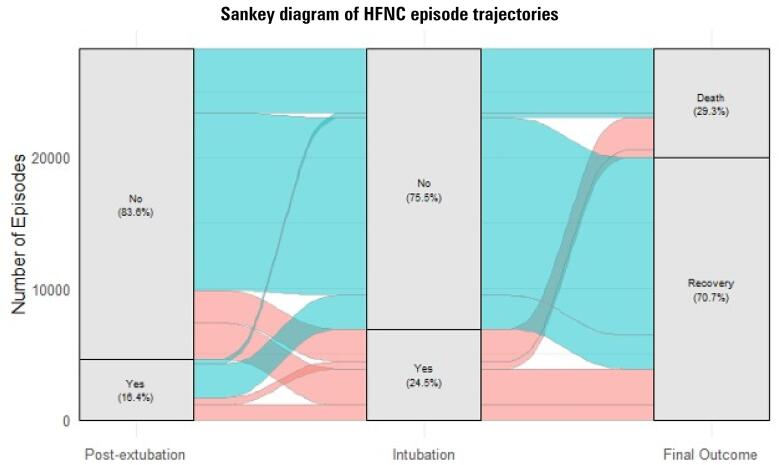
Sankey diagram illustrating the proportion of high flow nasal cannula episodes that were preceded by extubation in the prior 24 hours (left column), the proportion that were followed by intubation within 48 hours (central column), and the proportion that were followed by recovery or death (right column).

Overall, out of all 28,269 HFNC episodes, 6,916 (24%) were followed by intubation during the same hospitalization. 8,275 (29%) of all episodes occurred during a hospitalization ending in death or hospice discharge (Figure 2, Table 2S [Supplementary Material]). Of the 23,636 escalation episodes, 5,237 (22%) were followed by intubation during the same hospitalization, and 7,318 (31%) were during hospitalizations ending in death or hospice discharge (Table 2).

**Table 2:** High-flow nasal cannula outcomes stratified by key populations

		Escalation therapy (n = 23,636)	Post extubation (n = 4,633)	Followed within 48 hours by MV (n = 3,315)
Followed by intubation*		5,237 (22)	1,679 (36)	3,315 (100)
Dead or hospice discharge		7,318 (31)	957 (21)	1,509 (46)
Treated in an ICU		19,772 (84)	4,630 (100)	3,262 (98)
Hospital LOS (days)		12.3 (7.01 - 23.2)	21.7 (12.8 - 37.4)	20.1 [10.5 - 35.2]
ICU LOS (days)		6.57 (3.33 - 12.8)	10.8 (5.88 - 18.9)	11.4 [5.98 - 20.3]
Discharge location if discharged alive				
	Acute inpatient rehab	1,060 (4)	541 (12)	232 (7)
	Assisted living	4,099 (17)	1,024 (22)	532 (16)
	Home	9,865 (42)	1,912 (41)	740 (22)
	Transfer to another hospital	926 (4)	95 (2)	218 (7)
	Other	343 (1)	93 (2)	86 (3)

MV - mechanical ventilation; ICU - intensive care unit; LOS - length of stay.

* Followed by intubation during the same hospitalization at any point after the high flow nasal cannula episode ended. Continuous variables summarized as median [interquartile range], categorical variables as n (%); "Assisted Living" includes skilled nursing facilities and long-term acute care hospitals; "Other" includes discharge to shelter, psychiatric hospital.

Patients initiated on HFNC by a surgical service compared with a medical service were significantly more likely to be intubated within 48 hours (OR 1.32; 95%CI 1.18 - 1.47) but less likely to die (OR 0.50; 95%CI 0.44 - 0.58), even adjusted for patient demographics, severity of illness, code status, and admitting hospital type. The mean proportion of episodes that ended in intubation increased slightly over time between 2017 and 2024, from 15.0% to 20.8% (p = 0.008), but there were no meaningful changes in the proportions ending in death or hospice discharge.

Of escalation episodes that were followed by intubation, the median duration of HFNC prior to intubation was 10.9 [3.80 - 29.9] hours, and the median subsequent duration of intubation was 5.15 [2.42 - 11.0] days. Of intubations 70% occurred within 24 hours of HFNC, 85% within 48 hours, and 91% within 72 hours. Escalation episodes that were followed by intubation were significantly more likely to end in death or hospice discharge compared to those not followed by intubation (46% *versus* 28%; p < 0.001).

**Figure 3:**
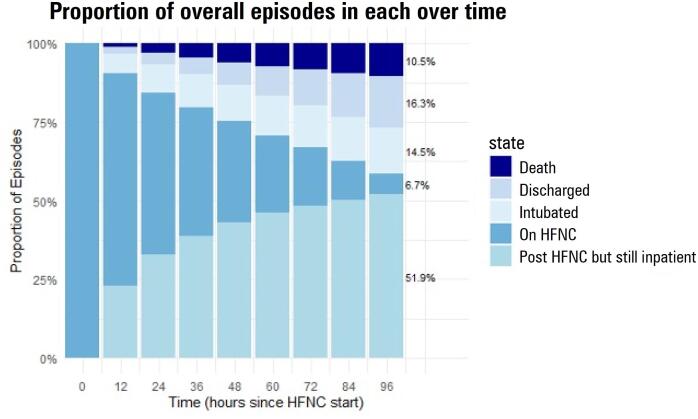
Stacked bar plot illustrating the relative proportions of high flow nasal cannula episodes that ended in death, ended in discharge, ended in invasive mechanical ventilation, ended in ongoing inpatient care off of high flow nasal cannula, or remained ongoing, measured at 12-hour intervals between the time of high flow nasal cannula initiation and 96 hours after initiation.

Approached differently, 96 hours after HFNC was initiated as escalation therapy for the first time, only 1,086 (7%) were still on HFNC, 2,345 (15%) were intubated, 2,622 (16%) had been discharged alive, 1,698 (11%) were dead or discharged to hospice, and 8,369 (52%) were still in the hospital neither intubated nor on HFNC (Figure 3).

HFNC - high flow nasal cannula.

**Figure 4:**
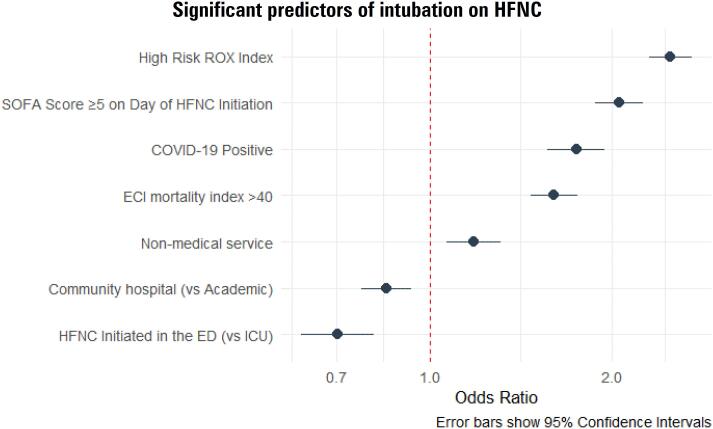
Forest-style plot illustrating the statistically significant results from a multivariable logistic regression model fit to assess the association between covariates of interest and odds of intubation in patients on high flow nasal cannula.

Factors associated with a greater odds of intubation within 48 hours, among those whose code status did not preclude intubation, included high risk ROX at HFNC initiation (OR 2.50; 95%CI 2.30 - 2.71), SOFA ≥ 5 (OR 2.05; 95%CI 1.88 - 2.25), COVID-19 positive status (OR 1.74; 95%CI 1.56 - 1.95), Elixhauser comorbidity weighted mortality index > 40 (OR 1.61; 95%CI 1.47 - 1.75), and admission to a non-medical service (1.18; 95%CI 1.07 - 1.31) (Figure 4). Patients in a community hospital (compared to an academic center) or in the ED (compared to the ICU) were significantly less likely to be intubated.

HFNC - high flow nasal cannula; SOFA - Sequential Organ Failure Assessment; ECI - Elixhauser Comorbidity Index; ED - Emergency Department; ICU - intensive care unit.

### Variation in high-flow nasal cannula across hospitals

In multivariable mixed-effects logistic regression, we found that 83.6% of the variation in mortality and 80.5% of the variation in intubation rates were attributable to measured patient and illness characteristics (Tables 3S and 4S - Supplementary Material). To translate these results into a clinical exemplar, for the same 65 year old White, Non-Hispanic Female with 8 Elixhauser comorbidities, "Full Code" code status, admitted to a medical service for COVID-19 negative respiratory failure and escalated to HFNC, with a SOFA of 5 and a high-risk ROX on the day of escalation, predicted intubation rate would range from 30.3% (95%CI 27.8 - 41.3%) to 38.9% (95%CI 28.3 - 41.2%) and mortality would range from 36.4% (95%CI 33.9 - 43.0%) to 40.7% (95%CI 33.9 - 42.9%), depending on admitting hospital.

## DISCUSSION

In this study, we characterized the current landscape of HFNC use in a multi-hospital U.S. system. We found that most HFNC use was escalation therapy in medical patients with hypoxemic respiratory failure. High-flow nasal cannula initiation occurred throughout the hospital, including outside formal critical care areas, and a quarter of patients were at high risk of failure at initiation. Duration of HFNC episodes varied, with a median duration of slightly more than a half-day, but with a tenth of patients spending over 3 days on HFNC. Approximately a quarter of all HFNC episodes end in intubation, and a third in death, but mortality rates were close to 50% in patients intubated after HFNC. Patient factors contributed far more to variability in HFNC outcomes than hospital factors, but variability not attributable to measurable patient factors was present.

### Relationship to previous work

High-flow nasal cannula is, in principle, a general supportive therapy for hypoxemia, with utility across a wide range of settings; this is the first study to confirm, on a system-wide scale, the heterogeneity of patients, contexts, and locations in which clinicians reach for HFNC.^(32)^ These findings raise important considerations for researchers and clinicians. As research on this topic proliferates (as of mid-June 2025, Clinicaltrials.gov registered 183 completed and 49 recruiting randomized control trials (RCTs) involving HFNC in adults), it will be important to consider the real world contexts in which research efforts will have the most impact and to reflect on the best approaches to studying a widely available tool with few barriers to initiation by clinicians.^(33)^

From a clinical perspective, the short median duration of HFNC therapy prior to intubation suggests a need for robust and reliable early risk assessment tools to serially assess response to HFNC at the bedside.^(34)^ The ROX, one of the few validated tools to predict HFNC failure, performs best 12 hours after HFNC initiation,^(29,30)^ at which point about half of patients in this study have either already been intubated or liberated from HFNC. Further, these tools may need to be implementable on the wards, where HFNC is frequently initiated despite practice guidelines suggesting it should be restricted to closely monitored settings.^(16)^ Further work evaluating the conditions of HFNC initiation on the wards and non-critical care providers’ decision-making surrounding HFNC is warranted.

An important ongoing clinical practice question that has proven challenging to answer is the optimal timing of intubation in patients with acute lung injury requiring respiratory support.^(27,35-39)^ We found that the duration of HFNC prior to intubation varied by admitting service type, hospital type, and unit type, even after adjusting for patient characteristics, suggesting that unmeasured clinical practice patterns may confound the relationship between HFNC duration and outcomes. Notably, studies that raise concerns about the harms of longer HFNC duration prior to intubation have defined "long duration" as 24 to 72 hours or more.^(26,28,40-43)^ We found that three-quarters of intubations following HFNC occur by 24 hours, and 90% by 72 hours, suggesting the importance of evaluating potential harms earlier. Promisingly, there may be sufficient variability in clinical practice that target trial emulation may be feasible to establish a causal link between duration and outcome or may justify the equipoise for further randomized clinical trials.^(39,44,45)^

Additionally, our study found lower intubation rates in patients treated in community hospitals compared with those in academic centers within our system, and these differences were not attributable to measured patient or illness characteristics. Plausible mechanisms for this finding include differences in ICU availability, staffing, comfort with HFNC use, and practices regarding thresholds to intubate patients. A full exploration of the mechanisms underlying this difference is an important area for future investigation.

We note that while recent large HFNC RCTs have excluded patients with code statuses limiting resuscitation,^(10,11)^ this population makes up 13% of patients on HFNC in our hospital system. Moreover, it is known that important decisions about goals of care are often avoided until a moment of crisis,^(46,47)^ and that it is often difficult for patients to accurately forecast their preferences until they become critically ill.^(48,49)^ These considerations raise the question of whether HFNC could be a pivotal moment for motivated goals-of-care discussions before more invasive and difficult-to-reverse therapies are initiated.^(50)^

This study differs from other recent cohort studies. First, it is the most extensive study describing HFNC use in the post-COVID-19 pandemic era. The pandemic had meaningful impacts on many aspects of critical care practice,^(51,52)^ but how it influenced clinical approaches to the management of hypoxemia is unknown. Although the use of certain therapies, such as prone positioning, increased during the pandemic and then returned to pre-pandemic levels,^(53)^ our results suggest sustained increased use of HFNC in the post-pandemic era. The extent to which this absolute increase in HFNC use is attributable to increasing numbers of respiratory failure cases treated over time versus to a difference in the proportional use of HFNC compared with other treatment modalities due to changes in clinician preferences or recent guidelines requires further study; specifically, the reasons for the relatively greater increase in post-extubation therapy *versus* escalation therapy is an area in need of investigation. Second, we approached our study of HFNC agnostic of a comparison group, such as NIPPV or a specific hospital setting. In clinical practice, there is great variety in decision-making about when, where, and why HFNC is initiated, and understanding general rates of outcomes such as intubation and mortality, regardless of how an episode of HFNC began, is informative.

### Limitations

Our study has limitations. First, this was a retrospective study of cases in which HFNC was used, but it is difficult to say whether HFNC was always required. It has been demonstrated by Yarnell et al. that substantial variability exists in clinicians’ beliefs about when intubation is necessary;^(54)^ similarly, the decision to initiate HFNC likely differs across clinicians. While trials of respiratory support have relied upon stringent definitions of intubation eligibility in order to mitigate inappropriate variability due to practice differences,^(37,38,55,56)^ when using retrospective EHR data, it is challenging to isolate the causal effects of HFNC initiation or duration compared to other modes of support (e.g., earlier intubation or NIPPV). We have therefore avoided causal language.

Second, it remains difficult to extract the clinical indication for an intervention such as HFNC from EHR data. As a result, our analyses are not adjusted for the specific cause of respiratory failure. Additionally, our data do not allow for distinctions in the clinical reasoning for post-extubation HFNC use (prophylactic use to prevent reintubation *versus* rescue for extubation failure) nor for the identification of cases in which HFNC was used solely for symptom relief. Third, there is no standard definition of a single "episode" of HFNC, and there is likely heterogeneity in how this is defined by trialists as compared to clinicians. Fourth, our study period included time before, after, and during the COVID-19 pandemic, and while we adjusted for patients’ COVID-19 status, we could not adjust for other unmeasured differences in practice patterns resulting from the pandemic. Other factors that may be of interest but are not assessed within this study include HFNC flow rate, HFNC delivered temperature, and the use of sedating medications. Finally, this study included only one hospital system, and there may be significant practice variation worldwide.

## CONCLUSION

In this study, we found that high-flow nasal cannula use occurs in a wide range of settings and contexts throughout the hospital. In absolute numbers, the use of high-flow nasal cannula has increased between 2017 and 2025. While most patients who started on high-flow nasal cannula recovered, those who progressed to intubation had a graver prognosis. We also found evidence of variability in practice due to unmeasured factors across our hospital system. There are several areas in need of further in depth, likely mixed methods, study including the development of tools which can be used shortly after high-flow nasal cannula initiation for reevaluation of clinical status and risk of intubation, the system and practice level factors which impact high-flow nasal cannula duration prior to intubation, the use of high-flow nasal cannula outside of critical care spaces, and the use of high-flow nasal cannula as a trigger for timely conversations with patients about preferences regarding life sustaining therapies.

## Data Availability

The data cannot be made publicly available. They are available for reanalysis upon execution of a duly completed data use agreement, as they contain protected health information.
