# Supplementary material for: Patterns and outcomes of real-world high-flow nasal cannula use: a multi-hospital retrospective cohort study
Source: Crit Care Sci. 2026 Feb 20;38:e20260366. doi: 10.62675/2965-2774.20260366 (PMC13124109; doi:10.62675/2965-2774.20260366)
Supplement: SUPPLEMENTARY MATERIAL [file 2965-2774-ccsci-38-e20260366-suppl01.pdf]

Critical Care Science
https://doi.org/10.62675/2965-2774.20260366

Patterns and outcomes of real-world high-flow nasal cannula use: a multi-hospital retrospective cohort study

Diana C. Bouhassira[1], Chad H. Hochberg[1], Sarina K. Sahetya[1], Ann Parker[1], Khyzer B. Aziz[2], Li Yan[1], Theodore John Iwashyna[1]

SECTION 1S - ADDITIONAL METHODS INFORMATION

Data construction

Patient demographic data, including age, sex, race, ethnicity, and primary language, were extracted from the electronic health record (EHR). Race, ethnicity, and primary language are self-reported in accordance with local hospital practices. Baseline Elixhauser Comorbidity Index (ECI) weighted mortality and readmission indices were calculated using EHR ICD-10 code data and previously published weighting methods.[1] Most recent code status prior to the high-flow nasal cannula (HFNC) episode and following code status were extracted from the EHR.[2]

Illness severity was measured using the Sequential Organ Failure Assessment (SOFA) Score and the ROX Index for Intubation (ROX), both calculated from physiologic and laboratory data as previously described.[3,468 (35.6%)] SOFA scores were calculated for the day of admission and the day of initiation of a HFNC episode; missing data were imputed as zero, consistent with standard practice. SOFA scores were calculated excluding the neurologic subscale because of concerns about the reliability and availability of the input data from chart extraction in our hospital system at present. ROX score was categorized as ≥ 5 (low) or < 5 (high) risk.[5] Coronavirus disease-2019 (COVID-19) status during hospitalization was assessed from ICD-10 billing code for COVID-19.

All time variables, including time of HFNC initiation, time HFNC was stopped, ICU admission and discharge times, hospital admission and discharge times, and time of death, were extracted in seconds and subsequently converted to hours or 24-hour periods ("days"). HFNC episode duration was calculated as the difference between HFNC start and end time and evaluated as a continuous variable (hours).

Hospitalization characteristics identified from the EHR included admitting hospital, unit type (intensive care unit [ICU], intermediate care unit [IMC], emergency department [ED], procedural/labor & delivery suite, or wards), admitting clinical service (medical, surgical, or other), and discharge location. Hospital and ICU length of stays were calculated using hospital and ICU admission start and end times, respectively.

Statistical analysis

Proportions were compared using chi-square tests. Continuous variables were compared using Student's t-tests or one-way analysis of variance (ANOVA) for normally distributed data and Wilcoxon rank-sum or Kruskal-Wallis tests for non-normally distributed data.

Variation across hospitals

Mixed-effects logistic regression models adjusting for patient and illness characteristics included fixed effects for age, sex, race, ethnicity, ECI mortality index, number of ECI comorbidities, admission month, SOFA score the day of HFNC initiation, ROX index at the start of HFNC, prior code status, whether intubated was permitted, extubation in the previous 24 hours, COVID status, and responsible clinical service.

This is an open access article under the CC BY license (https://creativecommons.org/licenses/by/4.0/).

For each outcome, Model 1 included only a random-effects term for hospital, and Model 2 included fixed effects for patient and illness characteristics and a random-effects term for hospital. We calculated the intraclass correlation coefficient (ICC) using the conventional method with $\pi^2/3$ for the residual variance[6] and the proportional change in variance attributable to between-hospital random effects.[7] Confidence intervals were calculated using parametric bootstrapping.[8]

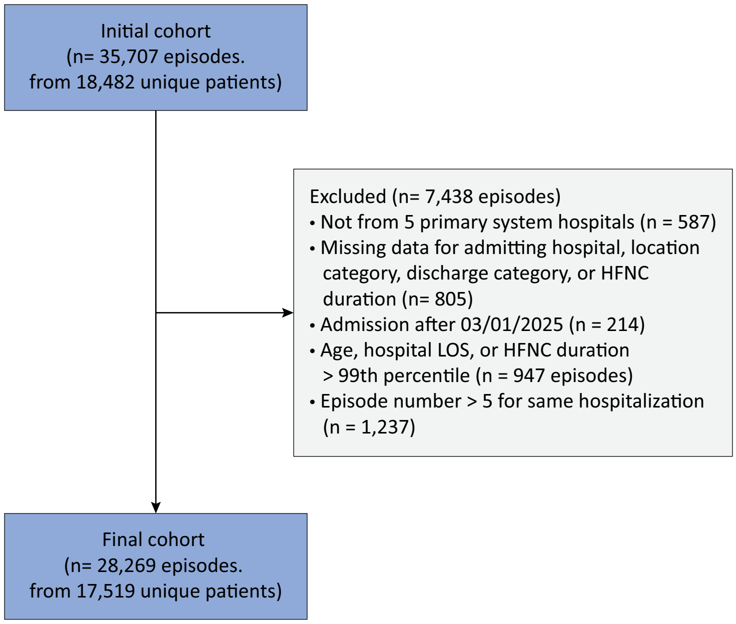

HFNC - high-flow nasal cannula; LOS - length of stay.

Figure 1S - Analytic cohort flow diagram.

Table 1S - Full results of multivariable linear regression with cluster-robust standard errors for factors associated with high-flow nasal cannula episode duration

	Estimate	CI lower	CI upper	p-value
Age >65	2.29	1.02	3.57	0.000
Male	0.04	-1.16	1.23	0.946
White race	1.06	-0.18	2.3	0.076
Hispanic ethnicity	5.23	1.83	8.63	0.000
Unknown ethnicity	-0.71	-2.76	1.35	0.504
ECI mortality index > 40	0.33	-1.13	1.79	0.634
# of ECI co-morbidities	-0.28	-0.45	-0.11	0.001
SOFA day of HFNC initiation > 4	-0.4	-1.67	0.86	0.525
SOFA day of admission > 4	-2.12	-3.35	-0.89	0.001
High risk ROX Index	-0.68	-2.06	0.7	0.306
COVID19 positive	12.07	10.03	14.11	0.000
Initiated in ED	-1.77	-6.31	2.78	0.415
Initiated in the Procedural area or L&D	1.43	-7.09	9.96	0.724
Initiated in IMC	8.32	3.84	12.81	0.000
Initiated on wards	9.55	7.15	11.96	0.000
Community hospital	1.47	0.13	2.81	0.023
Admission Month - 2	-0.51	-3.39	2.37	0.704
Admission Month - 3	-3.13	-5.89	-0.37	0.018
Admission Month - 4	-3.72	-6.39	-1.05	0.004
Admission Month - 5	-1.71	-4.53	1.1	0.187
Admission Month - 6	-2.11	-4.95	0.74	0.125
Admission Month - 7	-1.94	-4.78	0.91	0.153
Admission Month - 8	-0.78	-3.58	2.02	0.555
Admission Month - 9	-1.95	-4.73	0.82	0.141
Admission Month - 10	-0.83	-3.63	1.96	0.526
Admission Month - 11	0.6	-2.17	3.38	0.634
Admission Month - 12	-1.8	-4.39	0.79	0.136
Intubation permitted by code status	0.19	-1.69	2.08	0.833
Service - other	-0.43	-4.9	4.04	0.841
Service - surgical	-4.08	-5.52	-2.64	0.000

ECI - Elixhauser Comorbidity Index; SOFA - Sequential Organ Failure Assessment; IMC - intermediate care unit.

Table 2S - Descriptive table of intubation versus vital status

	Discharged alive n (%)	Discharged deceased or to hospice n (%)	
Not intubated after HFNC	16,111 (57.0)	5,242 (18.5)	Total not intubated: 21,353 (75.5)
Ever intubated after HFNC	3,883 (13.7)	3,033 (10.7)	Total intubated: 6,916 (24.5)
	Total discharged alive: 19,994 (70.7)	Total discharged deceased/hospice: 8,275 (29.3)	

HFNC - high flow nasal cannula.

Table 3S - Multivariable mixed-effects logistic regression model assessing hospital-level variance

Results of mixed-effects logistic regression models		Death	Intubation
Unadjusted model	Hospital-level variance	0.0412	0.0907
	ICC (hospital-level variance/total variance)	0.0123	0.0177
Adjusted with patient factors	Hospital-Level Variance	0.0067	0.0057
	ICC (hospital-level variance/total variance)	0.0020	0.0053
	Proportional change in variance (compared to the unadjusted model), %	83.7	80.5

Note that additional specifications of the methods used are available above.
The ICC for the empty model (Model 1) was 1.23%, suggesting 1.23% of variation in in-hospital mortality was attributable to differences between hospitals. Adding patient and illness characteristics (Model 2), the ICC decreased to 0.2% and hospital-level variance decreased by 83.7%, suggesting that 16.3% of between-hospital variability was not explained by the included patient or illness characteristics. The analogous analysis for intubation demonstrated that 1.77% of the variation in intubation was attributable to differences between hospitals, and 80.5% of the between-hospital variation was explained by patient and illness characteristics.

Table 4S - Characteristics of included hospitals

Hospital	Hospital type	# of hospital beds	# of IMC beds	# of ICU beds	ICU type	Average annual # of IMV patients*	Average # of ECI comorbidities†	Average ECI mortality Index‡	# of HFNC episodes (2017-2025)	Proportion of HFNC episodes intubated (%)	Proportion DNI prior to HFNC (%)
1	Academic	420	15	12<br>12<br>12<br>8<br>10	Medical<br>Coronary care<br>Surgical<br>Neurological<br>Burn	949	8 [5 - 11]	18 [3 - 36]	4,371	24.8	11.5
2	Academic	1,091	33	24<br>12<br>10<br>12<br>12<br>22<br>22	Medical<br>CCU<br>Oncological<br>SICU1<br>SICU2<br>Cardiothoracic<br>Neurological	2,264	8 [6 - 11]	27 [10 - 45]	13,035	28.3	7.8
3	Community	225	17	33	General	375	8 [5 - 10]	20 [5 - 38]	4,685	15.8	21.4
4	Community	230	20	18<br>18	General<br>General	687	7 [5 - 10]	23 [8 - 41]	4,279	25.3	15.8
5	Community	318	0	14	General	139	7 [5 - 10]	27 [8 - 47]	899	16.5	21.5

IMC - intermediate care unit; ICU - intensive care unit; IMV - invasive mechanical ventilation; ECI - Elixhauser comorbidity index; HFNC - high flow nasal cannula; DNI - do not intubate code status; CCU – cardiac care unit; SICU – surgical intensive care unit. *Average calculated over 2017-2025; † median [interquartile range] number of Elixhauser Comorbidities of patients in study cohort, stratified by hospital; ‡ median [interquartile range] ECI mortality index of patients in study cohort, stratified by hospital.

REFERENCES

1. van Walraven C, Austin PC, Jennings A, Quan H, Forster AJ. A modification of the Elixhauser comorbidity measures into a point system for hospital death using administrative data. Med Care. 2009;47(6):626-33.
2. Hochberg CH, Gersten RA, Aziz KB, Krasne MD, Yan L, Turnbull AE, et al. The real-world effect of early screening for palliative care criteria in a medical intensive care unit: an instrumental variable analysis. Ann Am Thorac Soc. 2025;22(2):247-54.
3. Roca O, Caralt B, Messika J, Samper M, Sztrymf B, Hernández G, et al. An index combining respiratory rate and oxygenation to predict outcome of nasal high-flow therapy. Am J Respir Crit Care Med. 2019;199(11):1368-76.
4. Brinton DL, Ford DW, Martin RH, Simpson KN, Goodwin AJ, Simpson AN. Missing data methods for intensive care unit SOFA scores in electronic health records studies: results from a Monte Carlo simulation. J Comp Eff Res. 2022;11(1):47-56.
5. Ricard JD, Roca O, Lemiale V, Corley A, Braunlich J, Jones P, et al. Use of nasal high flow oxygen during acute respiratory failure. Intensive Care Med. 2020;46(12):2238-47.
6. Merlo J, Chaix B, Ohlsson H, Beckman A, Johnell K, Hjerpe P, et al. A brief conceptual tutorial of multilevel analysis in social epidemiology: using measures of clustering in multilevel logistic regression to investigate contextual phenomena. J Epidemiol Community Health. 2006;60(4):290-7.
7. Seymour CW, Iwashyna TJ, Ehlenbach WJ, Wunsch H, Cooke CR. Hospital-level variation in the use of intensive care. Health Serv Res. 2012;47(5):2060-80.
8. Loy A, Korobova J. Bootstrapping Clustered Data in R using lmeresampler. R J. 2023;14(4):103-20.
